# An Algorithm for Finding Biologically Significant Features in Microarray Data Based on *A Priori* Manifold Learning

**DOI:** 10.1371/journal.pone.0090562

**Published:** 2014-03-03

**Authors:** Zena M. Hira, George Trigeorgis, Duncan F. Gillies

**Affiliations:** Department of Computing, Imperial College London, London, United Kingdom; University of Illinois-Chicago, United States of America

## Abstract

Microarray databases are a large source of genetic data, which, upon proper analysis, could enhance our understanding of biology and medicine. Many microarray experiments have been designed to investigate the genetic mechanisms of cancer, and analytical approaches have been applied in order to classify different types of cancer or distinguish between cancerous and non-cancerous tissue. However, microarrays are high-dimensional datasets with high levels of noise and this causes problems when using machine learning methods. A popular approach to this problem is to search for a set of features that will simplify the structure and to some degree remove the noise from the data. The most widely used approach to feature extraction is principal component analysis (PCA) which assumes a multivariate Gaussian model of the data. More recently, non-linear methods have been investigated. Among these, manifold learning algorithms, for example Isomap, aim to project the data from a higher dimensional space onto a lower dimension one. We have proposed *a priori* manifold learning for finding a manifold in which a representative set of microarray data is fused with relevant data taken from the KEGG pathway database. Once the manifold has been constructed the raw microarray data is projected onto it and clustering and classification can take place. In contrast to earlier fusion based methods, the prior knowledge from the KEGG databases is not used in, and does not bias the classification process—it merely acts as an aid to find the best space in which to search the data. In our experiments we have found that using our new manifold method gives better classification results than using either PCA or conventional Isomap.

## Introduction

In machine learning as the dimensionality of the data rises, the amount of data required to provide a reliable analysis grows exponentially. Richard E. Bellman referred to this phenomenon as the “curse of dimensionality” when considering problems in dynamic optimisation [1]. A popular approach to this problem of high-dimensional datasets is to search for a projection of the data onto a smaller number of variables (or features) which preserves the information as much as possible. Microarray data is typical of this type of small sample problem. Each data point (microarray) can have up to 50,000 variables (gene probes) and processing a large number of data points involves high computational cost for obtaining a statistical significant result [2].

In the last ten years, machine learning techniques have been investigated in microarray data analysis. Several approaches have been tried in order to: (i) distinguish between cancerous and non-cancerous samples; (ii) classify different types of cancer and (iii) to identify subtypes of cancer that may progress aggressively. All these investigations are seeking to generate biologically meaningful interpretations of complex datasets that are sufficiently interesting to drive follow-up experimentation.

Many methods have been implemented for extracting only the important information from the microarrays thus reducing their size. The simplest is feature selection, in which the number of gene probes in an experiment is reduced by selecting only the most significant according to some criterion such as high levels of activity. A number of investigations of this kind have been used to examine breast cancer [3], [4], while other studies use different techniques such as support vector machines recursive feature elimination [5], leave-one-out calculation sequential forward selection, gradient-based-leave-one-out gene selection, recursive feature addition and sequential forward selection [6].

Feature extraction methods have also been widely explored. The most widely used method is principal component analysis (PCA) and many variations of it have been applied as a way of reducing the dimensionality of the data in microarrays [7]–[11]. A supervised version of PCA was described in [12]. PCA however has an important limitation: it cannot capture non-linear relationships that often exists in data, especially in complex biological systems.

An approach to dimensionality reduction that can take into account potential non-linearity is based on the assumption that the data (genes of interest) lie on an embedded non-linear manifold which has lower dimension than the raw data space and lies within it. Algorithms based on manifold learning work well when the high dimensionality of the data sets is artificially high; although each point is defined by thousands of variables, it can be accurately characterised by just a few. Samples are drawn from a low-dimensional manifold that is embedded in a high-dimensional space [13]. A commonly used method of finding an appropriate manifold, Isomap [14], constructs the manifold by joining each point only to its nearest neighbours. Distances between points are then taken as geodesic distances on the resulting graph. Many variants of Isomap have also been used, for example Balasubramanian and Schwartz [15] presented a tree connected version which differs in the way the neighbourhood graph is constructed. The *k*-nearest points are found by constructing a minimum spanning tree using an *ε-*radius hypersphere. Isomap has been tried on microarray data with some very good results [16], [17]. Compared to PCA, Isomap was able to extract more structural information about the data.

We have been investigating a novel way of constructing the manifold which makes use of prior knowledge. Prior knowledge has previously been used in microarray studies [18]–[20] with the objective of improving the classification accuracy. Although several types of prior knowledge could have been used, we chose the information in the *KEGG* pathways database. KEGG (Kyoto Encyclopedia of Genes and Genomes) [21] is a collection of databases containing information on networks of molecular interaction in different organisms. It is widely believed that these lower level interactions can be seen as the building blocks of genetic systems, and can be used to understand high-level functions of the biological systems. KEGG pathways have been quite popular in network constrained methods which use networks to identify gene relations to diseases [22], [23]. Other studies have used protein-to-protein interaction (PPI) networks for the same purpose [24]. Gene Ontology (GO) terms are a popular source of prior knowledge since they describe known functions of genes [18]–[20], [25]. We chose the KEGG pathways in the hope that they will provide more information about the diseases related to the genes than the functionality provided by the more abstract GO terms.

Our method of building the manifold is as follows. In common with all previous methods we first build an affinity matrix from a set of microarrays. A gene-by-gene affinity matrix is a square matrix whose dimension is the same as the number of gene probes in the microarray data. The matrix is symmetric and each entry is a similarity measure (for example covariance) of the expression levels of the two genes that index it. We then fuse information from the KEGG pathways increasing the values in the affinity matrix for gene pairs with a strong relationship in KEGG. Next we apply a conventional manifold learning method to the fused affinity matrix to find the manifold. Having found the manifold of the gene probes we then project the raw data onto it so we can carry out classification experiments. This means that the KEGG pathway data is only involved in building the manifold. In contrast to previous data fusion approaches [26], the prior knowledge is only used to find a suitable space for representing the data. Classification algorithms are applied on the raw data alone, and are not biased by the prior knowledge. This ensures that the results are more specific to the biological content of the dataset under investigation.

## Results

**Figure 1:**
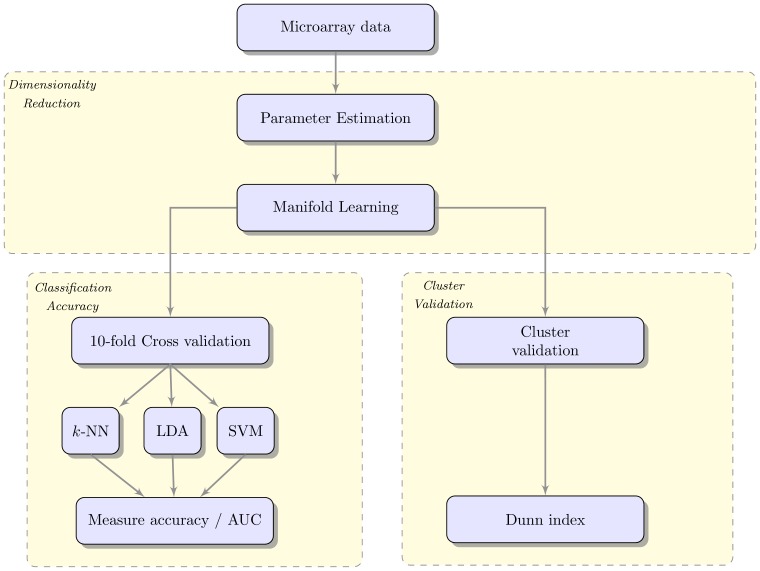
Evaluation benchmark. The 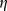 parameter is estimated and the resulting embedding is evaluated using cluster validation and cluster accuracy metrics.

To verify the effectiveness of our method we tested *a priori* manifold learning against the original Isomap algorithm and PCA. We used the Dunn Index which is a metric for evaluating the density and the structure of the clusters in the embedding. We also employed the *k*-Nearest Neighbours (*k*-NN), Support Vector Machines (SVMs) and Linear Discriminant Analysis (LDA) classifiers with 10-fold cross validation to test the accuracy of the model. Nine different types of cancer were used to evaluate the methods and we used a smaller dataset to visualise the results. The datasets are described in table 1. The evaluation scheme is shown in figure 1.

**Table 1:** Datasets Used.

Type Of Cancer	Number Of Samples	Number Of Genes
Breast cancer	344 cancer samples vs 1201 Other	10935
Colon cancer	286 cancer samples vs 1259 Other	10935
Kidney cancer	260 cancer samples vs 1285 Other	10935
Ovary cancer	198 cancer samples vs 1347 Other	10935
Lung cancer	126 cancer samples vs 1419 Other	10935
Uterus cancer	124 cancer samples vs 1421 Other	10935
Omentum cancer	77 cancer samples vs 1468 Other	10935
Prostate cancer	69 cancer samples vs 1476 Other	10935
Endometrium cancer	61 cancer samples vs 1484 Other	10935
Acute lymphoblastic leukaemia	19 B-Cell vs 8 T-Cell vs 10 Normal	5000

Description of the datasets used

### Internal Evaluation

#### Dunn Index

**Figure 2:**
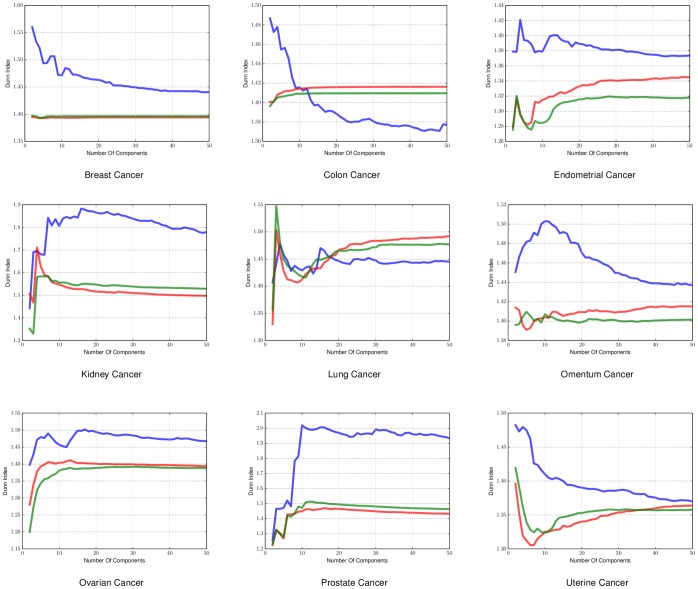
Dunn Index applied on sample-by-sample manifold for different cancers. The Dunn Index found using *a priori* manifold learning learning (Blue) compared with PCA (Green) and Isomap (Red) computed using the sample-by-sample affinity matrix.

**Figure 3:**
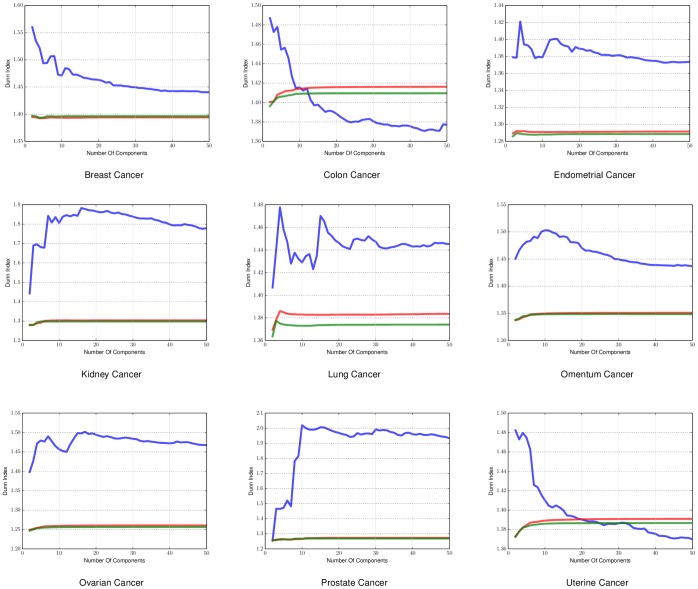
Dunn Index applied on gene-by-gene manifold for different cancers. The Dunn Index found using *a priori* manifold learning learning (Blue) compared with PCA (Green) and Isomap (Red) computed using the gene-by-gene affinity matrix.

The first metric we used to evaluate the density of the clusters is the Dunn Index. The Dunn Index is a way to measure the difference of the objects in a cluster with the mean of the same cluster. The higher the index value the better the state of the clusters. For our experiments the Dunn Index can indicate how well the resulting embedding separates the samples according to their label, since it uses the labels of each sample as the cluster indicators. In practice manifold learning does not create any clusters but if the embedding is done in a successful way many points will end up being next to each other, since the embedding is just a mapping from the original dataset to a different space. We ran this experiment for different dimensional embeddings (2 to 50 components) as the components we will end up using in the embedding is heavily dependent on the complexity of the data. We applied it on both sample-by-sample affinity matrices, shown in figure 2, and gene-by-gene affinity matrices shown in figure 3.

The results for the Dunn Index in sample-by-sample experiments in figure 2 and gene-by-gene experiments in figure 3 show that *a priori* manifold learning creates denser clusters in all cases except colon, uterine and lung cancer. From the graph induced from the colon dataset for both sample-by-sample and gene-by-gene experiments and the uterine dataset in the gene-by-gene experiments we can see that *a priori* manifold learning outperforms PCA and Isomap for embeddings with lower dimensions. Our goal is to create an embedding with as few components possible to represent the original high-dimensional data. For the lung dataset in the sample-by-sample experiments we need more samples to create a more accurate embedding.

### Ten fold cross-validation

To evaluate the accuracy of the embeddings we used the *k*-NN and LDA classifiers with ten fold cross validation to measure the accuracy of our method. In order to get the values we used the trapezoidal rule which approximates the definite integral of the plots. Results are shown in table 2 for sample-by-sample experiments and in table 3 for gene-by-gene experiments using *k*-NN. The corresponding results for LDA is shown in table 4 for sample-by-sample and in table 5 for gene-by-gene experiments. We have emphasised in bold the cases which *a priori* manifold learning outperforms the rest of the methods. It should be noted that the variance is small enough so we can compare the individual accuracies of the experiments safely. The variances for the *k*-NN classifier for the gene-by-gene experiments are shown in table 6 and for the sample-by-sample experiments in table 7. For the LDA the variance is shown in table 8 for the gene-by-gene experiments and in table 9 for the sample-by-sample experiments. We also demonstrate the accuracy error. The graphs can be found in Material S2. For the *k*-NN gene-by-gene experiments the graphs are shown in figure S1 and for the sample-by-sample in figure S2. For the Linear Discriminant Analysis gene-by-gene experiments graphs are shown in figure S3 and for the sample-by-sample in figure S4. In the LDA results *a priori* manifold learning outperforms PCA and Isomap for 6 out of 9 datasets. These are the same datasets for both sample-by-sample and gene-by-gene experiments. For the datasets that *a priori* manifold learning does not perform as good as the other two methods the problem might lie to the lack of a sufficient number of pathways in the KEGG database.

**Table 2:** 10 Fold Cross Validation Accuracy On Sample-by-Sample Transformation using *k*-Nearest Neighbours.

Type Of Cancer	A Priori Manifold Learning	Isomap	PCA
Breast cancer	0.806	0.863	0.879
Colon cancer	0.868	0.897	0.906
Kidney cancer	**0.937**	0.931	0.932
Ovary cancer	0.841	0.842	0.851
Lung cancer	0.902	0.911	0.917
Uterus cancer	0.891	0.890	0.891
Omentum cancer	**0.914**	0.912	0.912
Prostate cancer	**0.955**	0.954	0.954
Endometrium cancer	0.923	0.924	0.926

The results of 10-fold cross-validation on the dataset using sample-by-sample affinity matrices for PCA and Isomap. The *a priori* manifold learning method (which operates using a gene-by-gene affinity matrix) still provides comparable results with the other methods, while outperforming them in some of the cases. We have emphasised in bold the cases which *a priori* manifold learning outperforms the rest of the methods.

**Table 3:** 10 Fold Cross Validation Accuracy On Gene-by-Gene Transformation using *k*-Nearest Neighbours.

Type Of Cancer	*A priori* manifold learning	Isomap	PCA
Breast cancer	**0.806**	0.782	0.792
Colon cancer	**0.868**	0.834	0.834
Kidney cancer	**0.937**	0.900	0.903
Ovary cancer	**0.841**	0.834	0.838
Lung cancer	**0.902**	0.883	0.886
Uterus cancer	**0.891**	0.882	0.881
Omentum cancer	**0.914**	0.912	0.912
Prostate cancer	**0.955**	0.943	0.945
Endometrium cancer	**0.923**	0.922	0.922

The results of 10-fold cross-validation on the dataset using gene-by-gene affinity matrices for PCA and Isomap. The *a priori* manifold learning method clearly outperforms the other two. We have emphasised in bold the cases which *a priori* manifold learning outperforms the rest of the methods.

**Table 4:** 10 Fold Cross Validation Accuracy On Sample-by-Sample Transformation using Linear Discriminant Analysis.

Type Of Cancer	*A priori* manifold learning	Isomap	PCA
Breast cancer	0.890	0.901	0.912
Colon cancer	0.906	0.914	0.925
Kidney cancer	**0.956**	0.952	0.953
Ovary cancer	**0.871**	0.867	0.870
Lung cancer	0.935	0.938	0.941
Uterus cancer	**0.906**	0.900	0.905
Omentum cancer	**0.927**	0.923	0.924
Prostate cancer	**0.973**	0.972	0.972
Endometrium cancer	**0.937**	0.934	0.930

The results of 10-fold cross-validation on the dataset using gene-by-gene affinity matrices for PCA and Isomap. The *a priori* manifold learning method clearly outperforms the other two. We have emphasised in bold the cases which *a priori* manifold learning outperforms the rest of the methods.

**Table 5:** 10 Fold Cross Validation Accuracy On Gene-by-Gene Transformation using Linear Discriminant Analysis.

Type Of Cancer	*A priori* manifold learning	Isomap	PCA
Breast cancer	0.890	0.888	0.910
Colon cancer	0.906	0.914	0.924
Kidney cancer	**0.956**	0.911	0.954
Ovary cancer	**0.871**	0.945	0.870
Lung cancer	0.935	0.924	0.940
Uterus cancer	**0.906**	0.901	0.905
Omentum cancer	**0.927**	0.926	0.923
Prostate cancer	**0.973**	0.970	0.972
Endometrium cancer	**0.937**	0.932	0.930

The results of 10-fold cross-validation on the dataset using gene-by-gene affinity matrices for PCA and Isomap. The *a priori* manifold learning method clearly outperforms the other two. We have emphasised in bold the cases which *a priori* manifold learning outperforms the rest of the methods.

**Table 6:** 10 Fold Cross Validation Variance On Gene-by-Gene Transformation using *k*-Nearest Neighbours.

Type Of Cancer	A Priori Manifold Learning	Isomap	PCA
Breast cancer	32.09034e-5	37.52164e-5	35.38524e-5
Colon cancer	29.24537e-5	29.91476e-5	28.95183e-5
Kidney cancer	6.72999e-5	11.64989e-5	12.68591e-5
Ovary cancer	21.39207e-5	11.13463e-5	12.88114e-5
Lung cancer	14.09877e-5	5.26385e-5	3.13050e-5
Uterus cancer	13.01978e-5	3.44257e-5	5.51030e-5
Omentum cancer	2.54772e-5	0.80620e-5	0.80620e-5
Prostate cancer	2.34272e-5	6.79816e-5	4.34986e-5
Endometrium cancer	1.58922e-5	1.92059e-5	1.10440e-5

The results show that the variance of the cross validation is very small and thus we can safely compare the methods tested.

**Table 7:** 10 Fold Cross Validation Variance On Sample-by-Sample Transformation using *k*-Nearest Neighbours.

Type Of Cancer	A Priori Manifold Learning	Isomap	PCA
Breast cancer	32.09034e-5	27.91171e-5	18.32800e-5
Colon cancer	29.24537e-5	26.86585e-5	16.95718e-5
Kidney cancer	6.72999e-5	10.34294e-5	9.40982e-5
Ovary cancer	21.39207e-5	24.88867e-5	14.85025e-5
Lung cancer	14.09877e-5	14.62143e-5	12.39355e-5
Uterus cancer	13.01978e-5	16.97889e-5	18.60610e-5
Omentum cancer	2.54772e-5	2.79939e-5	2.12314e-5
Prostate cancer	2.34272e-5	2.07739e-5	2.17724e-5
Endometrium cancer	1.58922e-5	5.77868e-5	6.19262e-5

The results show that the variance of the cross validation is very small and thus we can safely compare the methods tested.

**Table 8:** 10 Fold Cross Validation Variance On Gene-by-Gene Transformation using Linear Discriminant Analysis.

Type Of Cancer	A Priori Manifold Learning	Isomap	PCA
Breast cancer	4.43639e-5	3.18558e-5	1.64494e-5
Colon cancer	3.97728e-5	1.79713e-5	5.60684e-5
Kidney cancer	6.28824e-5	2.24769e-5	2.73758e-5
Ovary cancer	2.94021e-5	3.21893e-5	3.50449e-5
Lung cancer	1.58082e-5	2.14339e-5	1.26192e-5
Uterus cancer	1.04442e-5	7.45783e-5	7.01667e-5
Omentum cancer	1.21062e-5	3.76439e-5	2.42125e-5
Prostate cancer	4.12092e-5	1.40641e-5	4.41161e-5
Endometrium cancer	1.14222e-5	1.62528e-5	8.67444e-5

The results show that the variance of the cross validation is very small and thus we can safely compare the methods tested.

**Table 9:** 10 Fold Cross Validation Variance On Sample-by-Sample Transformation using Linear Discriminant Analysis.

Type Of Cancer	A Priori Manifold Learning	Isomap	PCA
Breast cancer	4.43639e-05	2.70937e-5	1.62271e-5
Colon cancer	3.97728e-5	3.55322e-5	5.32299e-5
Kidney cancer	6.28824e-5	4.56036e-5	3.06760e-5
Ovary cancer	2.94021e-5	2.80513e-5	4.17136e-5
Lung cancer	1.58082e-5	1.97068e-5	1.47822e-5
Uterus cancer	1.04442e-5	4.53130e-05	7.25349e-5
Omentum cancer	1.21062e-5	9.24128e-5	2.14339e-5
Prostate cancer	4.12092e-5	1.25679e-5	2.42809e-5
Endometrium cancer	1.14222e-5	8.63512e-5	8.75652e-5

The results show that the variance of the cross validation is very small and thus we can safely compare the methods tested.

The sample-by-sample affinity matrix cannot be computed directly using *a priori* manifold learning since it needs the genes for constructing the affinity matrix therefore *a priori* manifold learning only operates on a gene-by-gene affinity matrix. For the GEMLeR dataset, the sample-by-sample affinity matrix has dimensions 1545 by 1545. This is the number of microarrays in the dataset. The gene-by-gene affinity matrix is 10935 by 10935 which is the number of gene probes in each microarray.

#### Receiver Operating Characteristic Curves

In addition we created the Receiver Operating Characteristic (ROC) curves to illustrate the ratio of true positives and false positive results. We have used three different classification methods for illustrating the effectiveness of *a priori* manifold learning.

#### *k* - Nearest Neighbours (*k*-NN)

**Figure 4:**
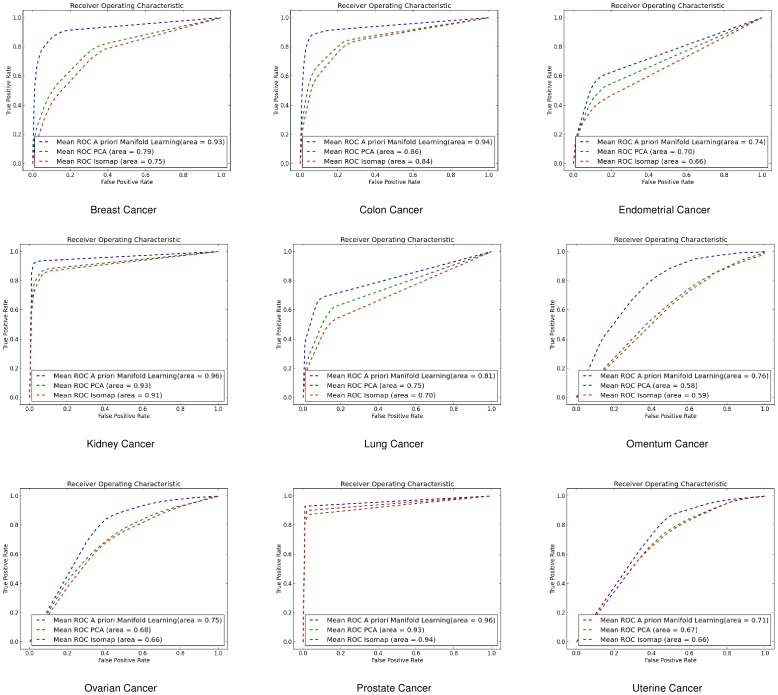
ROC curves for gene-by-gene affinity matrices using *k*-Nearest Neighbours. ROC curves found for *a priori* manifold learning (blue) compared with PCA (Green) and Isomap (Red) computed using the gene-by-gene affinity matrix and the *k*-NN classifier.

**Figure 5:**
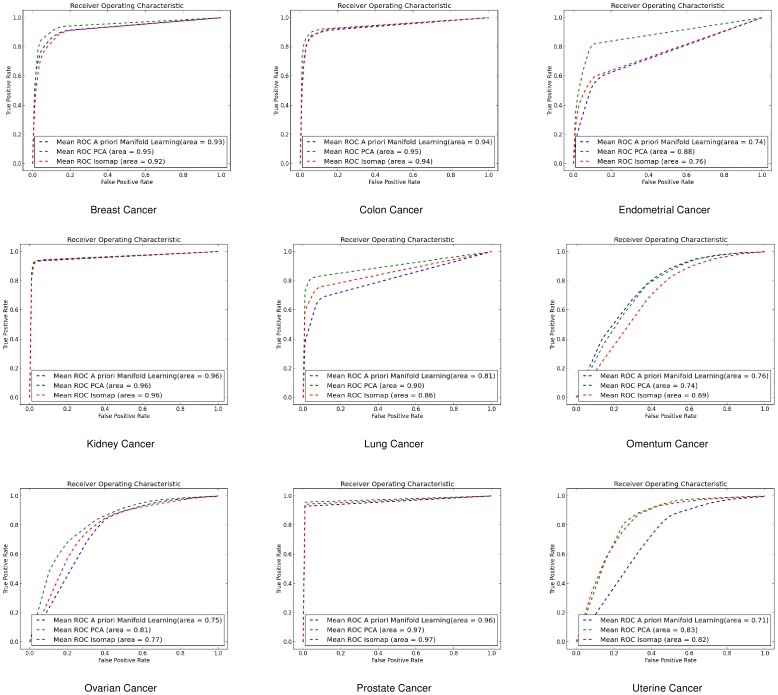
ROC curves for sample-by-sample affinity matrices using *k*-Nearest Neighbours. ROC curves found for *a priori* manifold learning (blue) compared with PCA (Green) and Isomap (Red) computed using the sample-by-sample affinity matrix and the *k*-NN classifier.

For the *k*-NN classifier the results we got for the ROC curves agree with the 10-fold cross validation results. *A priori* manifold learning performs better in all the gene-by-gene experiments as shown in figure 4, while in the sample-by-sample ones only performs better in one dataset as shown in figure 5

#### Support Vector Machines (SVMs)

**Figure 6:**
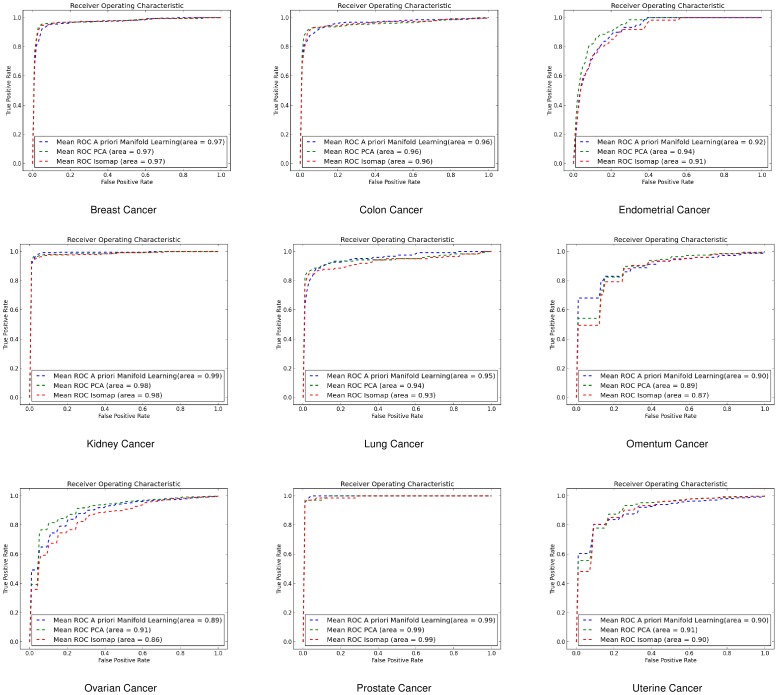
ROC curves for gene-by-gene affinity matrices using Support Vector Machines. ROC curves found for *a priori* manifold learning (blue) compared with PCA (Green) and Isomap (Red) computed using the gene-by-gene affinity matrix and the SVM classifier.

**Figure 7:**
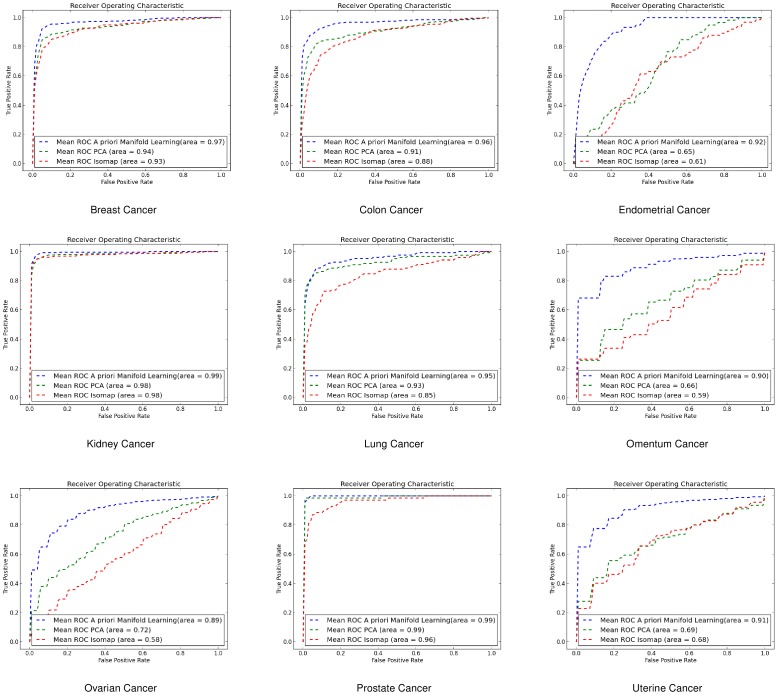
ROC curves for sample-by-sample affinity matrices using Support Vector Machines. ROC curves found for *a priori* manifold learning (blue) compared with PCA (Green) and Isomap (Red) computed using the sample-by-sample affinity matrix and the SVM classifier.

Using SVMs *a priori* manifold learning performs better in 7 out of 9 datasets for the gene-by-gene experiments (figure 6) while in the sample-by-sample experiments (figure 7) it performs better in all datasets.

#### Linear Discriminant Analysis (LDA)

**Figure 8:**
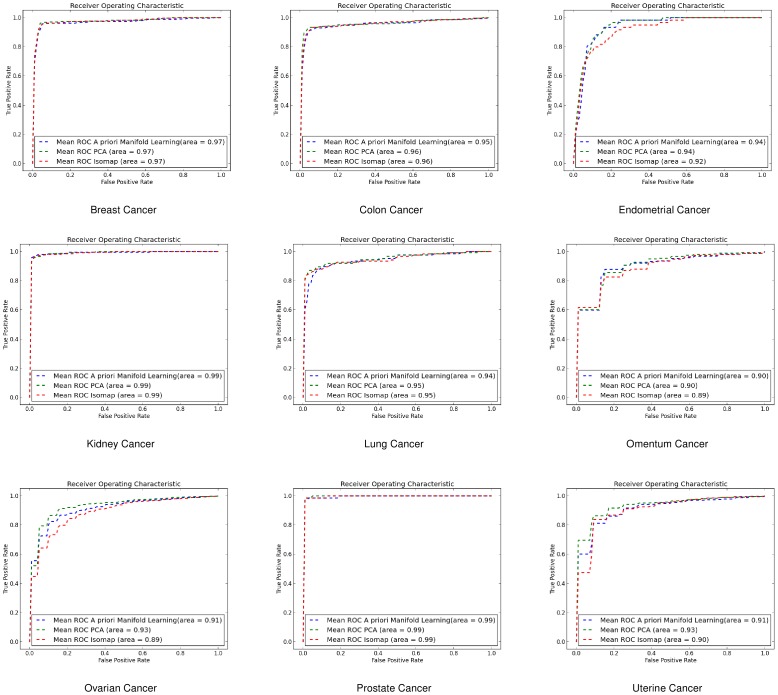
ROC curves for gene-by-gene affinity matrices using Linear Discriminant Analysis. ROC curves found for *a priori* manifold learning (blue) compared with PCA (Green) and Isomap (Red) computed using the gene-by-gene affinity matrix and the LDA classifier.

**Figure 9:**
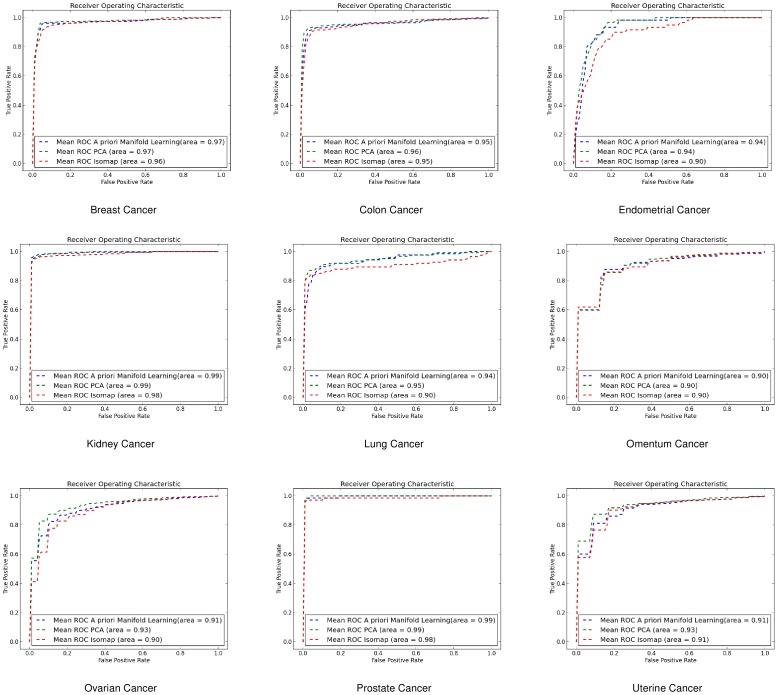
ROC curves for sample-by-sample affinity matrices using Linear Discriminant Analysis. ROC curves found for *a priori* manifold learning (blue) compared with PCA (Green) and Isomap (Red) computed using the sample-by-sample affinity matrix and the LDA classifier.

For the same purpose we also used LDA where for gene-by-gene experiments (figure 8) and sample-by-sample experiments (figure 9) *a priori* manifold learning performs better in 5 out of 9 datasets.

If we compare the ROC curves of the three different classifiers we can see that the *a priori* manifold learning gives consistent results for LDA and SVMs for both genes-by-gene and sample-by-sample experiments. However, the *k*-NN classifier seems to perform very well for the gene-by-gene experiments but not for the sample-by-sample ones. A possible explanation for this is that discriminant methods like SVMs and LDA use a data model computed from the whole data sets, and may therefore be more robust to noise and other artefacts. By contrast the *k*-NN classifier relies on the local distribution of the data, and could therefore be less effective particularly in small sample size problems.

**Figure 10:**
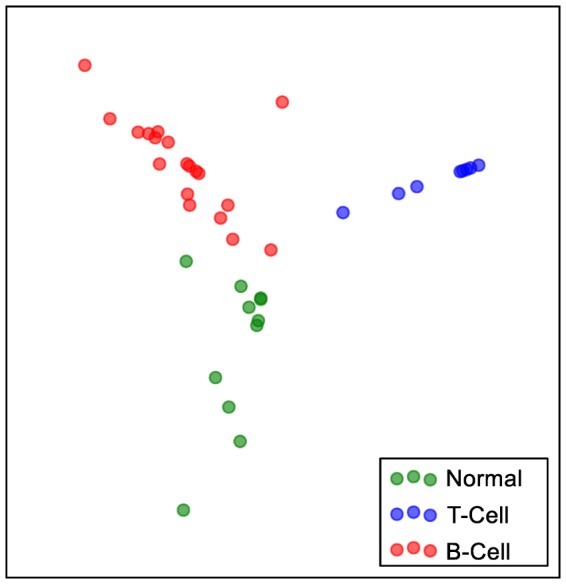
Leukaemia cell. Two dimensional manifold of the three different leukaemia cells. Clusters of the different cell types are formed and are easily distinguished in the lower dimensional space.

We used the Acute Lymphoblastic Leukaemia (ALL) dataset for leukaemia to demonstrate how the different cells were clustered. We have chosen the ALL dataset as it is simple enough to visualise and has been used before in [27] to demonstrate the clustering of the different types of cells in two dimensions. The embedding with the samples annotated with their true labels is found in figure 10.

## Discussion

Conventional manifold learning algorithms, such as Isomap, aim to project the microarray data to a lower dimensional space in which functionally different clusters are better separated. The lower dimensional space is a manifold (hypersurface) contained in the original data space and found from the local distribution of the data. A large representative dataset is used to compute the manifold. Our method provides a way of improving the way Isomap finds the *k*-nearest points and creates the neighbouring graph by utilising KEGG pathway information. The KEGG data is a form of prior knowledge which is better curated and more reliable than the microarray data. Once the manifold has been constructed the raw microarray data is projected onto it and clustering and classification can take place. We called this method *a priori* manifold learning and we compared it to the original Isomap and the PCA algorithms, since PCA is the most commonly used method for dimensionality reduction. By incorporating prior knowledge we argue that we are able to have less variable and more biologically significant clusters. Information taken from KEGG pathways is a way of decreasing the noise in the microarray experiments. We produced results using ten different datasets of cancer data, where we tried to distinguish among different types of cancers. Nine out of ten datasets are considered to be high dimensional.

The results were similar across the different datasets. In the first set of results, we showed, using the Dunn Index, that our algorithm is able to create denser clusters with objects that lie closer to the mean of the cluster with a small variance. *A priori* manifold learning produces more compact, well - separated clusters when compared with PCA and the original Isomap. In some cases *a priori* manifold learning performs better only for embeddings with a smaller number of components which is still useful since we are more interested in embeddings with a lower number of dimensions. There were also cases were the samples and the KEGG signatures were not enough for *a priori* manifold learning to perform better than PCA and Isomap.

Incorporating prior knowledge using KEGG pathways is not only limited to cancer data but it can be applied to a number of diseases that have KEGG signatures. This, along with the fact that the method does not require any other information, makes it easy to adapt to any kind of biological problem. Other studies [18]–[20] have used Gene Ontology (GO) terms instead of KEGG pathways. We believe that KEGG pathways carry more information when it comes to diseases rather than GO terms since GO terms mostly give information about the function of a gene.

When performing cross validation experiments both PCA and Isomap features can be computed using either the gene-by-gene affinity matrix or the sample-by-sample affinity matrix. The latter is a square matrix with dimension equal to the number of microarrays used in the experiment. Each entry represents the similarity (or distance) between the corresponding pair of microarrays. It is considerably smaller than the gene-gene matrix and consequently more robust to noise. *A priori* manifold learning can only be computed using the gene-by-gene affinity matrix. This is because the prior knowledge extracted from the KEGG data base is in the form of similarities between gene pairs. Our results show that both PCA and Isomap perform better using the sample-by-sample affinity matrix. *A priori* manifold learning on average performs better in all cases when using the LDA and SVM classifiers. It does not do so well in classification experiments where PCA and Isomap are computed using the sample-by-sample affinity matrix using the *k*-NN classifier. In this case there is no significant difference between the three formulations. A possible reason for this is that both LDA and SVM classifiers create a model of the underlying classes, but *k*-NN is a parametric method which depends on the local distribution of the data, and consequently may be more susceptible to noise.

Overall we see that *a priori* manifold learning produces better formed clusters than either PCA or Isomap, and also performs better in classification experiments using either SVM or LDA methods. One of the drawbacks of the method is that it has only been formulated using the gene-by-gene affinity matrix, and this makes it more susceptible to noise than methods that can be computed directly on a sample-by-sample affinity matrix. Consequently a current direction of further work is to investigate methods whereby prior knowledge can be used in a sample-by-sample formulation. We are also investigating ways in which we can make the prior knowledge more specific to the particular type of cancer under investigation. By doing so we hope to make inroads into the harder problem of recognising subtypes of a cancer that will progress aggressively.

## Materials and Methods

In this paper we present a method which incorporates manifold learning along with a novel approach for estimating the neighbourhood graph. The cluster validation and accuracy measures, along with the original Isomap algorithm and PCA were implemented using the *sklearn* [28] package for Python.

### Manifold Learning - Isomap

The manifold learning algorithm is used for non-linear dimensionality reduction [29]. Manifold learning generally works by embedding inputs from a higher dimensional space in a lower one while preserving their characteristics. It assumes that all data points are lying close to or on a manifold and it can be thought as a generalised principal components analysis (PCA) that can capture non-linear relations. Isomap, [14] short for Isometric Mapping, was one of the first approaches to manifold and is an extension to *Kernel PCA*. The Isomap algorithm works as follows:

1. Determine the neighbours: For all points in a fixed radius, find the *k* nearest points (*k* - Isomap) or the closest points based on distance (ϵ-Isomap)
2. Construct the neighbourhood graph: Points are connected to each other if they are *k* nearest points away with the edge length set to their Euclidean distance.
3. Find the shortest path between all the nodes on the graph using a graph algorithm (*Dijkstra* or *Floyd-Warshall*) to construct the matrix of pairwise geodesic distances between different points.
4. Construct the lower dimensionality mapping. This is the same procedure as classical MDS. Generally another matrix 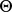 is constructed using:
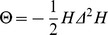
(1)where 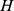 is the centering matrix:
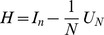
(2)where 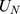 is an 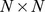 matrix of 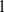
's;
     

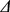
 is the matrix of geodesic distances;
     and 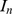 is the identity matrix of size *n*
5. Calculate the eigenvalues of 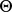: Let 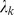 be the 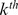 eigenvalue and 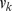 be the 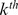 eigenvector. We construct the 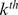 component of the embedding 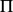 by setting it to 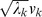.

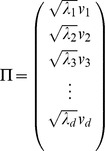

#### A priori Manifold Learning

**Figure 11:**
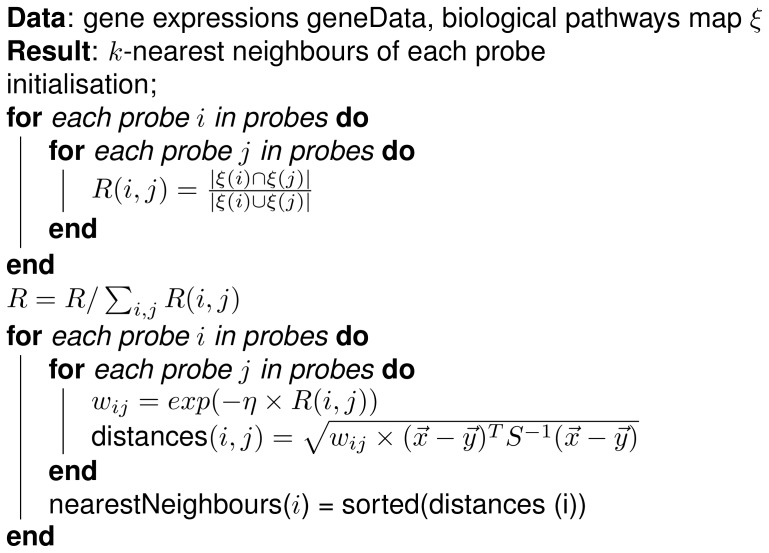
Calculation of the *k*-Nearest points of the manifold. First the Jaccard coefficient is calculated, the the Mahalanobis distances among the genes and the weights.

Biological pathways are usually directed graphs with labelled nodes and edges representing associations of genes participating in a biological process. These interactions can help in understanding the underlying processes in different organisms as well as their contribution to diseases. Some of the interactions include regulation of gene expression, transmission of signals and metabolic processes. It is not yet clear as to why and how these interactions came to exist and what other, if any, external factors contribute to them. When it comes to machine learning, information from the pathways can be used as prior knowledge for either feature selection or dimensionality reduction of the original data set. For our implementation, KEGG pathways are used as a way to weight the distance between the gene to gene interactions. Genes that share a greater number of common pathways should have more probability in being closer together when it comes to clustering. The metric we have used in weighting the distances was based on the method for feature selection [30]. This method works by assigning weights on the different features so that the more important ones play a greater role in the equation. By exploiting the use of these weights we can modify the classical *k* nearest points algorithm using the weighted Mahalanobis shown in equation (6) as a distance metric for determining which points of the original data space are close to one another. The algorithm to find the *k*-Nearest points works as follows:

1. Given a pair of probes the Jaccard coefficient is used to evaluate the similarity of pathways they share together. This index coined by Paul Jaccard [31] is a statistic commonly used for comparing similarity and diversity of sample sets shown in equation (3).
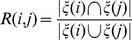
(3)where 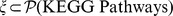.
2. The distance metric selected to calculate the gene-to-gene distance was the Mahalanobis distance. It is measured using the correlations between two datasets.
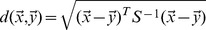
(4)where 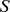 is the covariance matrix.
3. The weights equation is shown in equation (5)
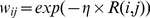
(5)where 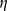 a learning parameter and 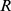 is the Jaccard coefficient. The learning parameter 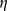 is a way of minimising and maximising the influence of any given feature in the dataset. When 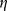 is large the changes in the dataset are exponentially reflected on the weights. They way the parameter 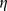 affects the results is shown in Material S1 in figure S5.
4. The weights along with the Mahalanobis distance are expressed as:
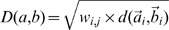
(6)The algorithm is shown in figure 11

#### Geodesic matrix and eigenvalues

The shortest paths 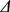 are found using either the Dijkstra [32] or Floyd-Warshall algorithm [33]. Dijkstra's algorithm is usually preferred since it is faster and the weights are non-negative. The Isomap mapping is done by calculating the eigenvalues of 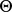 as shown in equation (1). If the mapping has been calculated from the gene to gene affinity matrix we denote it as 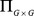. The corresponding eigenvalue basis for the sample-by-sample affinity matrix 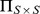 can be found by multiplying 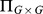 by the original data.



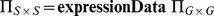
(7)


### Cluster evaluation methods

#### *k*-fold Cross Validation

To evaluate the results *k*-fold cross-validation [34] was used, where 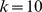. The embedding produced gets partitioned in 10 subsets, one of them is used for validation and the other 9 are used as the training data. The process is repeated 10 times so that every subset is used as validation exactly once. The results are averaged along 10 times and a single estimation is produced.

#### Support Vector Machines

A Support Vector Machine [35] is a classifier defined by a separating hyperplane. Given labelled training data, the algorithm outputs an optimal hyperplane which classifies new examples. Given a labelled training set 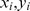 where 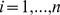 SVMs can find a solution to the following optimisation problem:
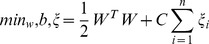
(8)


#### Linear Discriminant Analysis

Linear discriminant analysis [36] works by finding a linear combination of features which characterises or separates two or more classes if the likelihood ratios are less than a threshold 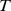 such that:
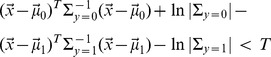
(9)assuming that the conditional probability density function 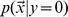 and 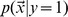 are normally distributed with mean 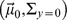 and covariance 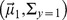.

#### Dunn Index

The Dunn Index is an internal evaluation metric for clusters [37]. Internal evaluation means that it only depends on the data of the cluster itself, mainly by considering better the clusters with little variance. It is defined as:
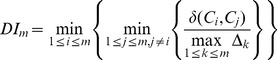
(10)where 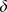 is the distance metric between the cluster 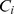 and 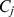 and 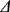 is
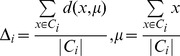
(11)and it computes the distance of all points from the mean.

### Pathway Robustness

**Figure 12:**
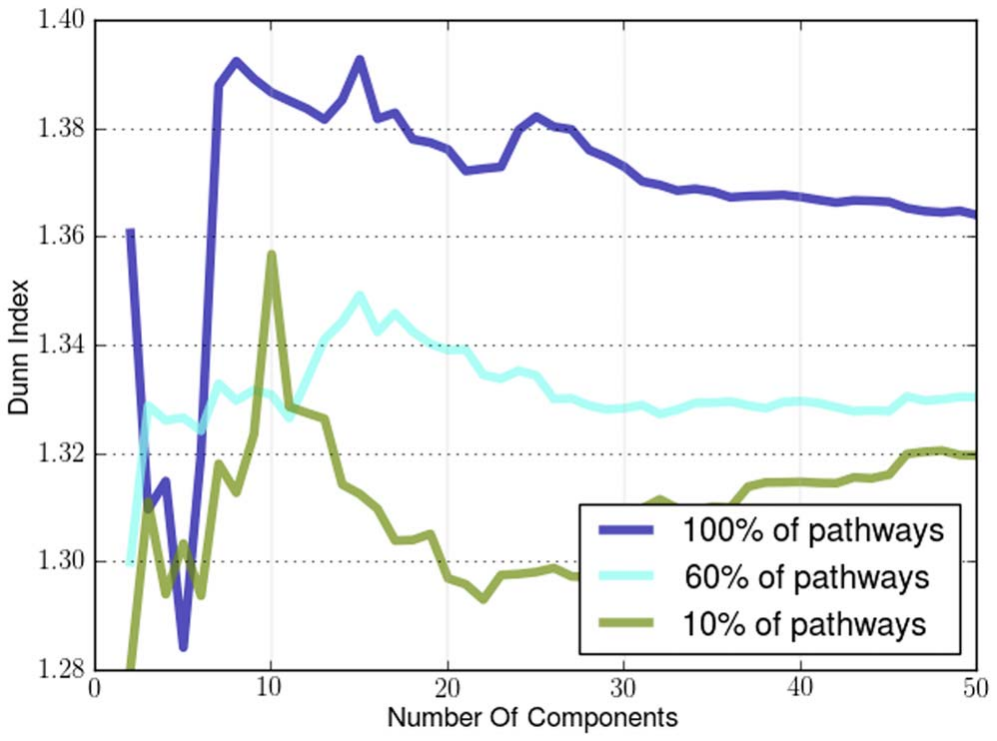
Pathway Robustness (Endometrium). A plot of the Dunn Index with different percentages of pathways.

**Figure 13:**
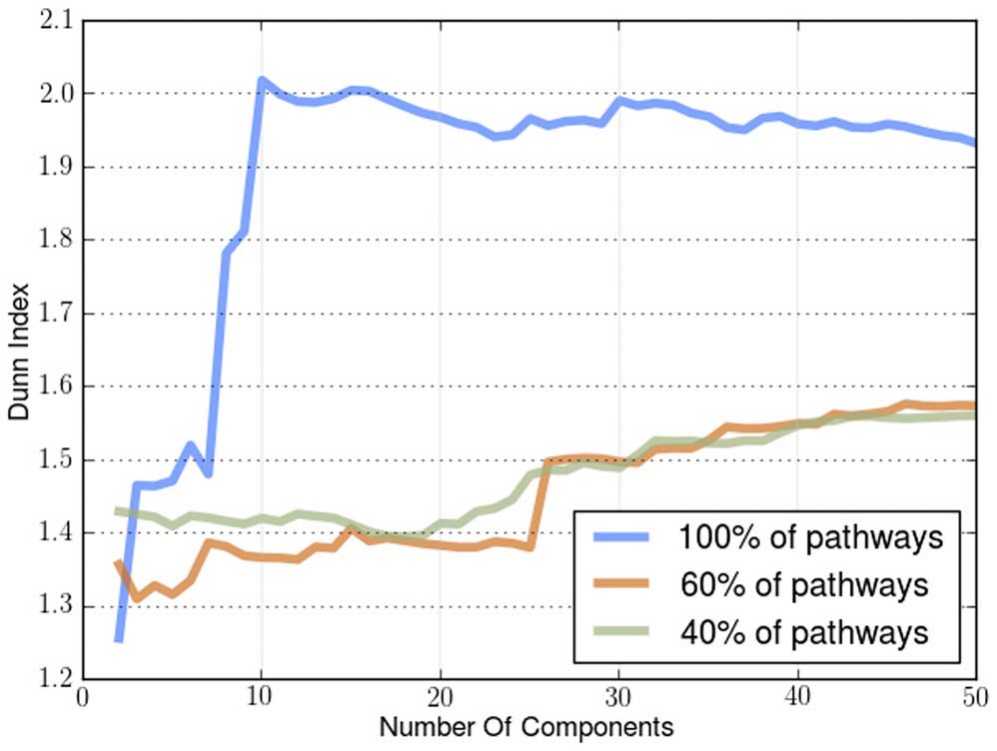
Pathway Robustness (Prostate). A plot of the Dunn Index with different percentages of pathways.

**Figure 14:**
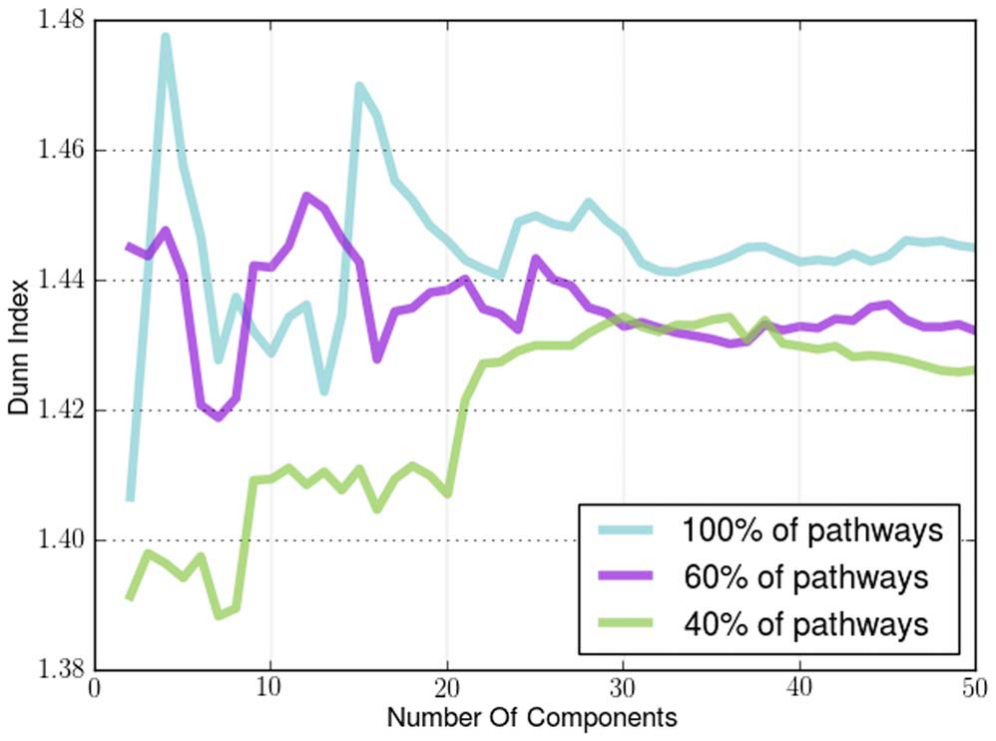
Pathway Robustness (Lung). A plot of the Dunn Index with different percentages of pathways.

**Figure 15:**
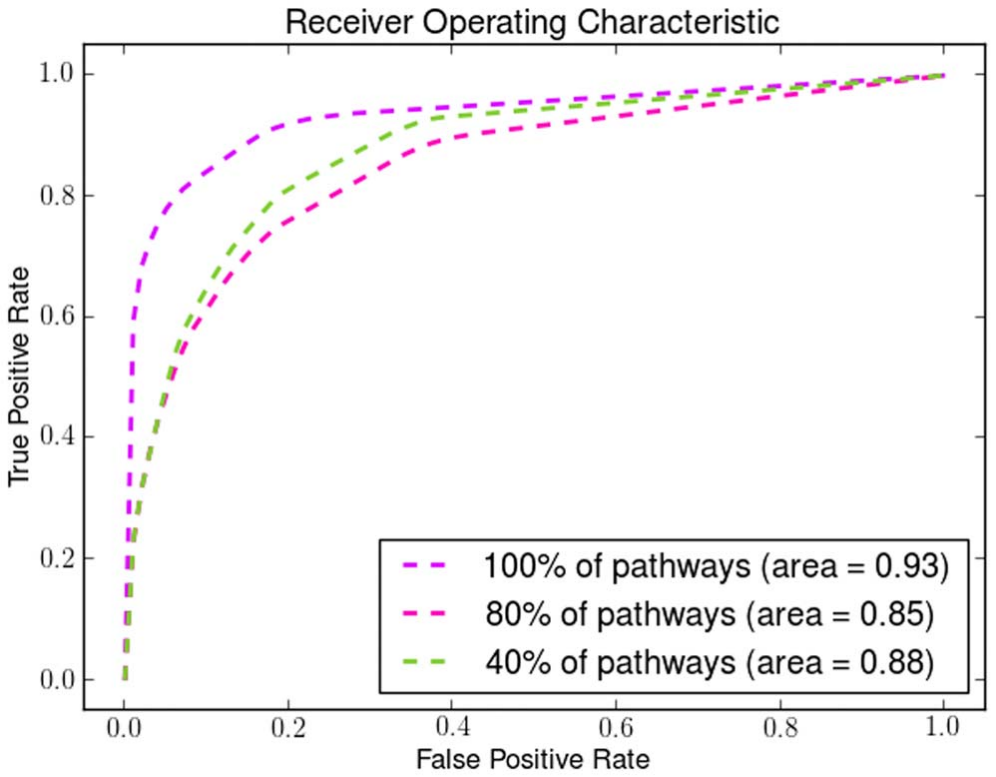
Pathway Robustness (Breast). A plot of ROC curves with different percentages of pathways.

**Figure 16:**
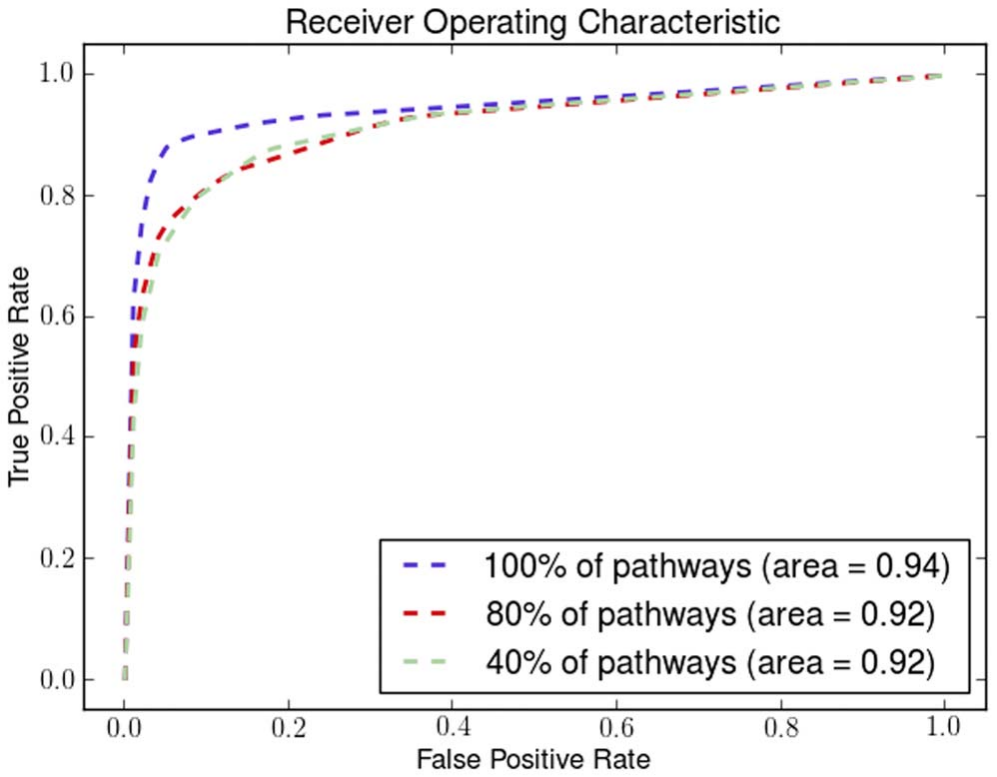
Pathway Robustness (Colon). A plot of ROC curves with different percentages of pathways.

**Figure 17:**
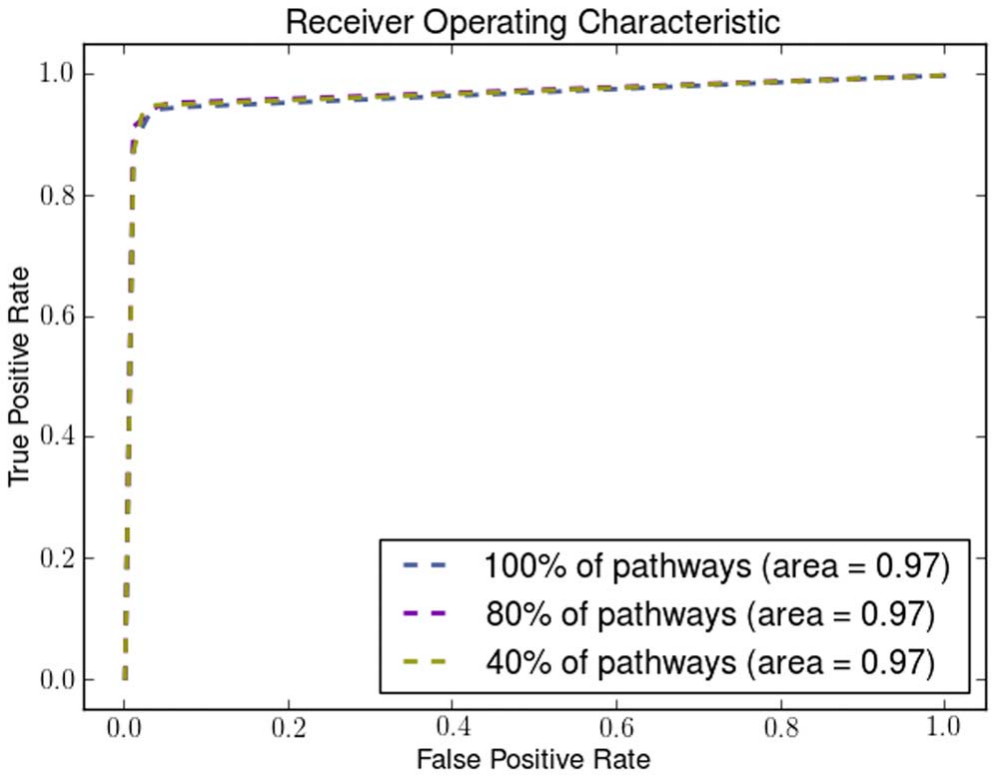
Pathway Robustness (Kidney). A plot of ROC curves with different percentages of pathways.

**Figure 18:**
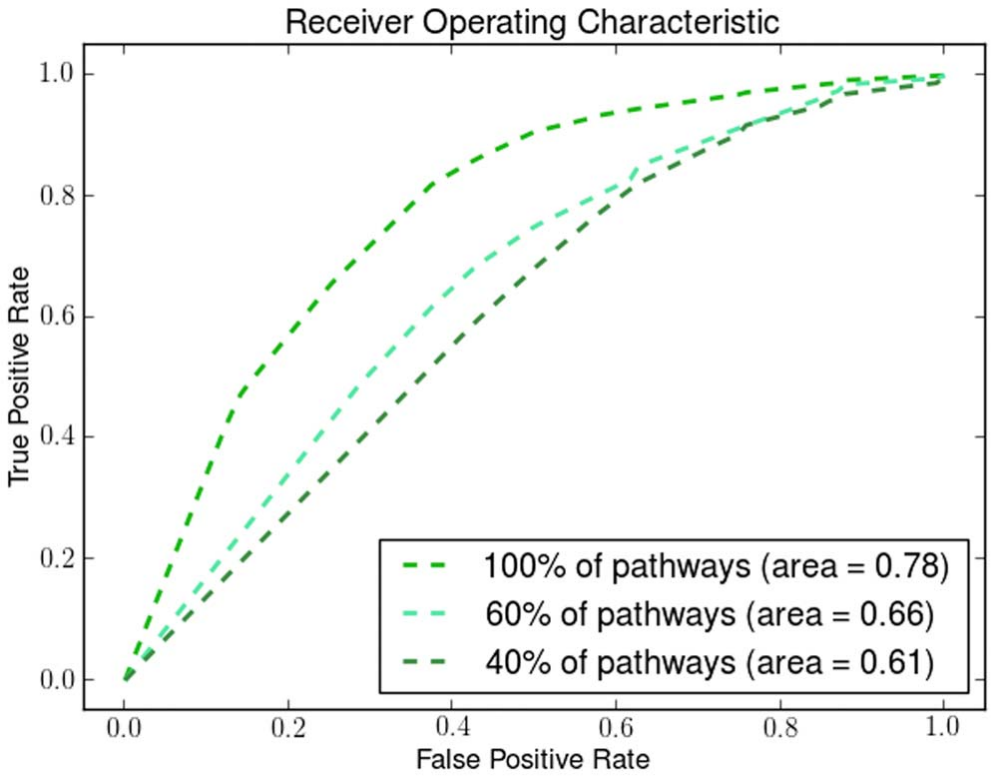
Pathway Robustness (Omentum). A plot of ROC curves with different percentages of pathways.

**Figure 19:**
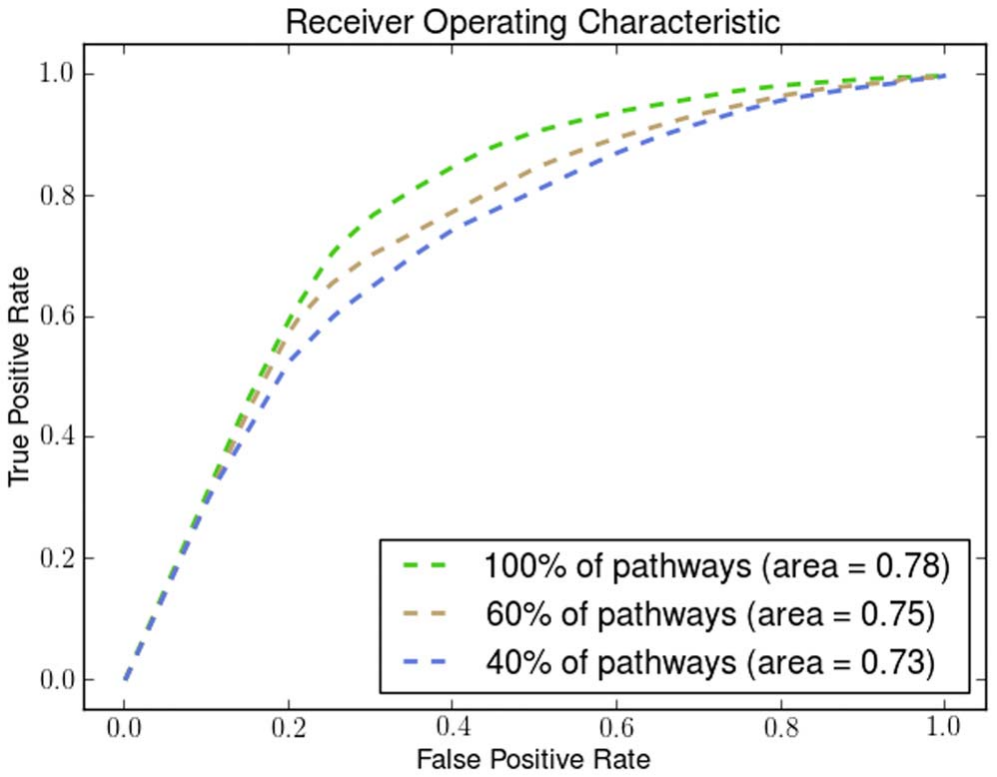
Pathway Robustness (Ovary). A plot of ROC curves with different percentages of pathways.

We demonstrate the robustness and the effectiveness of using pathways by removing pathways using a uniform distribution with different probabilities. By removing a percentage of the KEGG pathways in different runs of the algorithm we show how the number of pathways affects its performance. We show how the Dunn Index is affected in the Endometrium (figure 12), Prostate (figure 13) and Lung (figure 14) datasets. We also show how the ROC curves are affected for Breast in figure 15, Colon in figure 16, Kidney in figure 17, Omentum in figure 18 and Ovary in figure 19.

### Datasets

To test our *a priori* Manifold Learning method we used two different types of datasets.

1. GEMLeR, provides a collection of gene expression datasets that can be used for benchmarking gene expression oriented machine learning algorithms. Each of the gene expression samples in GEMLeR came from a large publicly available repository. GEMLeR was mainly preferred as:
  - The processing procedure of tissue samples is consistent
  - The same Affymetrix microarray assay platform is used (Affymetrix GeneChip U133 Plus 2.0)
  - There is large number of samples for different tumour types
  - Additional information is available for combined genotype-phenotype studies
2. Acute lymphoblastic leukaemia (ALL) is a form of leukaemia characterised by excess lymphoblasts. There are two main types of acute leukaemia: T-cell ALL and B-cell ALL. T-Cell acute leukaemia is aggressive and progresses quickly but is more common in older children and teenagers. B-Cell ALL leukaemia [38] is another type of ALL, originated in a single cell and characterised by the accumulation of blast cells that are phenomenologically reminiscent of normal stages of B-cell differentiation.

Information on the contents of the datasets is shown in table 1.

### Execution Times

Our algorithm takes approximately 45 minutes for each embedding which is the same as the original Isomap algorithm. PCA is however a lot faster since is only takes ten minutes to fit the data and create an embedding. This is because PCA is linear while *a priori* manifold learning and Isomap are non-linear methods and they need more time to fit the data.

## Supporting Information

Figure S1 **Accuracy with variance for all nine datasets for gene-by-gene affinity matrices** ***k*** **-Nearest Neighbours.** Accuracy with variance calculated for *a priori* manifold learning (blue) compared with PCA (Green) and Isomap (Red) computed using the gene-by-gene affinity matrix and the *k*-NN classifier.
(TIFF)

Figure S2 **Accuracy with variance for all nine datasets for sample-by-sample affinity matrices using** ***k*** **-Nearest Neighbours.** Accuracy with variance calculated for *a priori* manifold learning (blue) compared with PCA (Green) and Isomap (Red) computed using the sample-by-sample affinity matrix and the *k*-NN classifier.
(TIFF)

Figure S3 **Accuracy with variance for all nine datasets for gene-by-gene affinity matrices using Linear Discriminant Analysis.** Accuracy with variance calculated for *a priori* manifold learning (blue) compared with PCA (Green) and Isomap (Red) computed using the gene-by-gene affinity matrix and the LDA classifier.
(TIFF)

Figure S4 **Accuracy with variance for all nine datasets for sample-by-sample affinity matrices using Linear Discriminant Analysis.** Accuracy with variance calculated for *a priori* manifold learning (blue) compared with PCA (Green) and Isomap (Red) computed using the sample-by-sample affinity matrix and the LDA classifier.
(TIFF)

Figure S5
**Endometrium Cancer.** How the 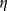 value affects the value for the Dunn Index.(TIFF)

Material S1
**The** 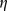 **Value.** We show how the 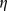 value improves the Dunn Index. The 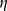 value selected for the embedding of the Endometrial cancer was 19000. It is the value with the highest Dunn Index as shown in figure S5.(PDF)

Material S2 **Accuracy Variance.** We present the error bars with one standard deviation of uncertainty for the 10-fold cross validation with a *k*-NN classifier in figure S2 for the sample-by-sample affinity matrix and in figure S1 for gene-by-gene affinity matrix. For Linear Discriminant Analysis the gene-by-gene errorbars are shown in figure S3 and for the sample-by-sample experiments in figure S4.
(PDF)
