# Supplementary figures and images for: An Algorithm for Finding Biologically Significant Features in Microarray Data Based on A Priori Manifold Learning
Source: PLoS One. 2014 Mar 3;9(3):e90562. doi: 10.1371/journal.pone.0090562 (PMC3940899; doi:10.1371/journal.pone.0090562)

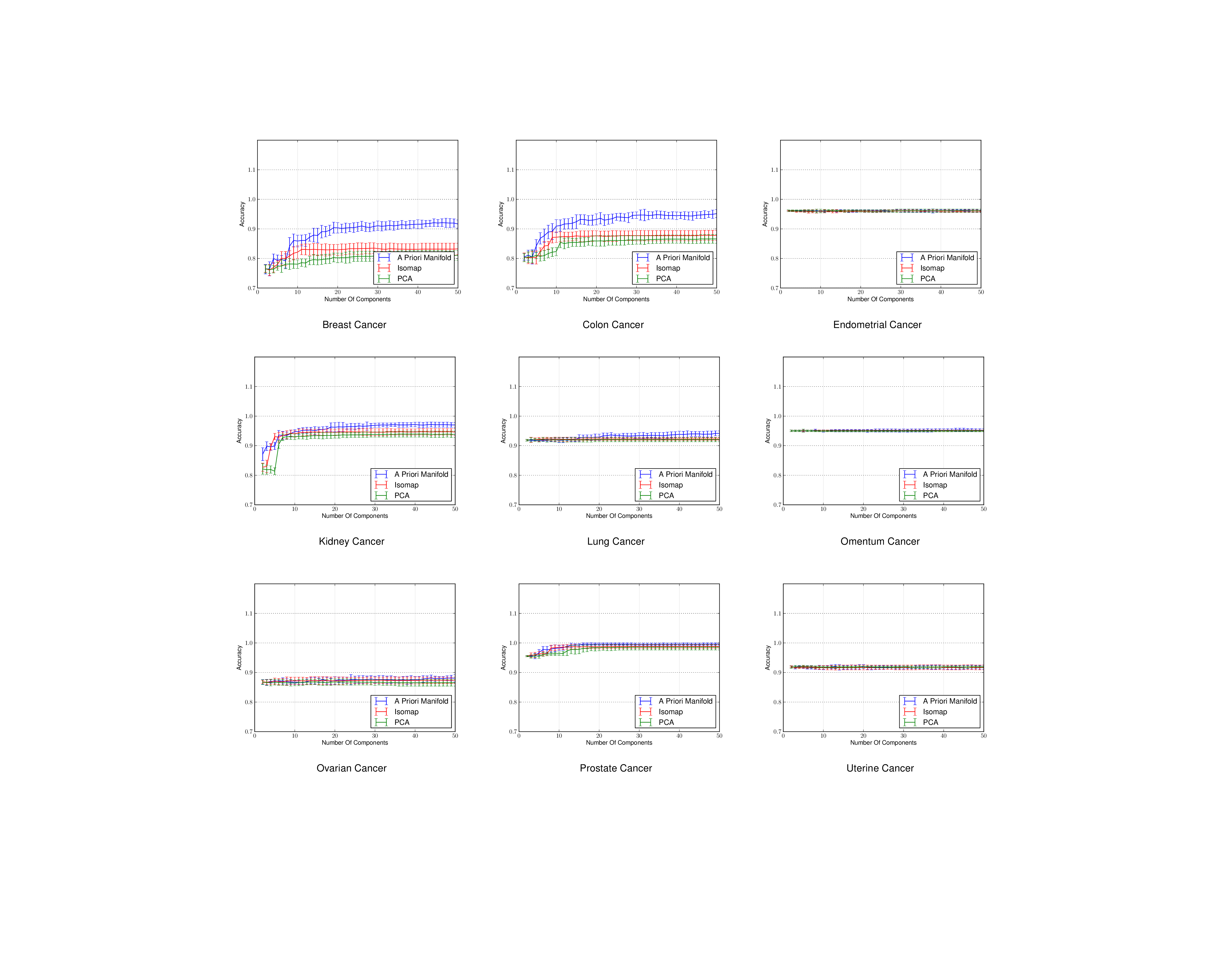

Supplement: Figure S1 — Accuracy with variance for all nine datasets for gene-by-gene affinity matrices k -Nearest Neighbours. Accuracy with variance calculated for a priori manifold learning (blue) compared with PCA (Green) and Isomap (Red) computed using the gene-by-gene affinity matrix and the k-NN classifier. (TIFF) [file pone.0090562.s001.tiff]

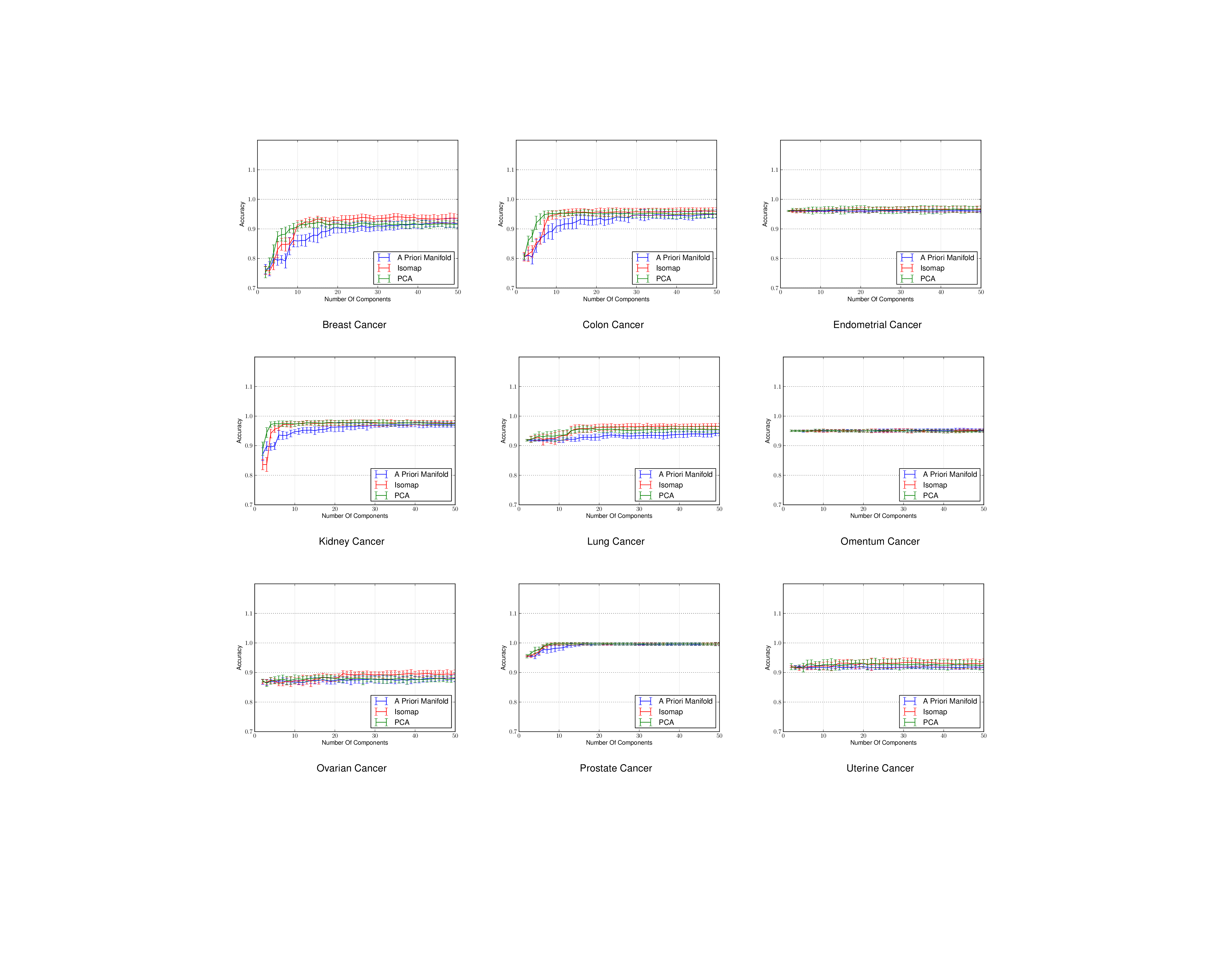

Supplement: Figure S2 — Accuracy with variance for all nine datasets for sample-by-sample affinity matrices using k -Nearest Neighbours. Accuracy with variance calculated for a priori manifold learning (blue) compared with PCA (Green) and Isomap (Red) computed using the sample-by-sample affinity matrix and the k-NN classifier. (TIFF) [file pone.0090562.s002.tiff]

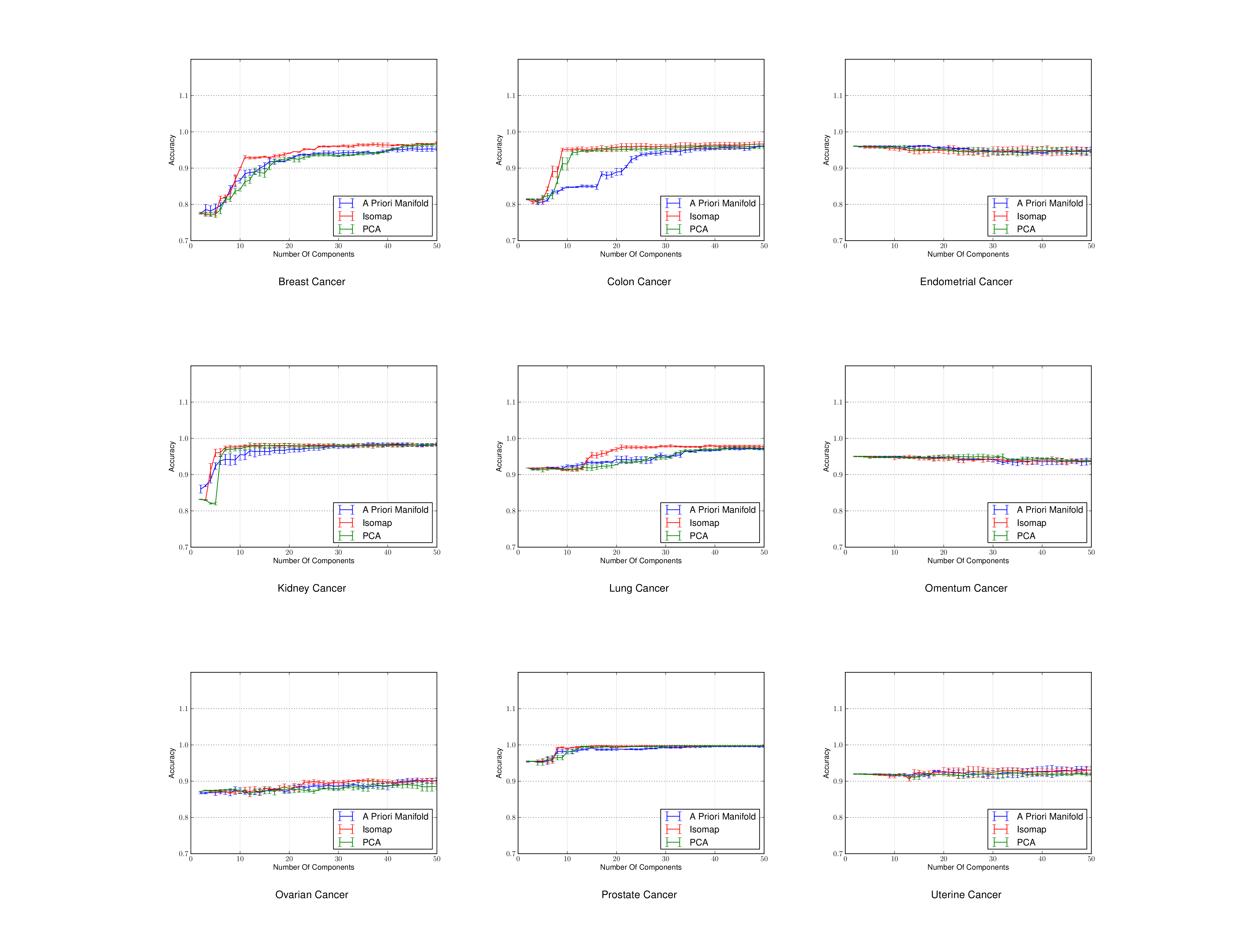

Supplement: Figure S3 — Accuracy with variance for all nine datasets for gene-by-gene affinity matrices using Linear Discriminant Analysis. Accuracy with variance calculated for a priori manifold learning (blue) compared with PCA (Green) and Isomap (Red) computed using the gene-by-gene affinity matrix and the LDA classifier. (TIFF) [file pone.0090562.s003.tiff]

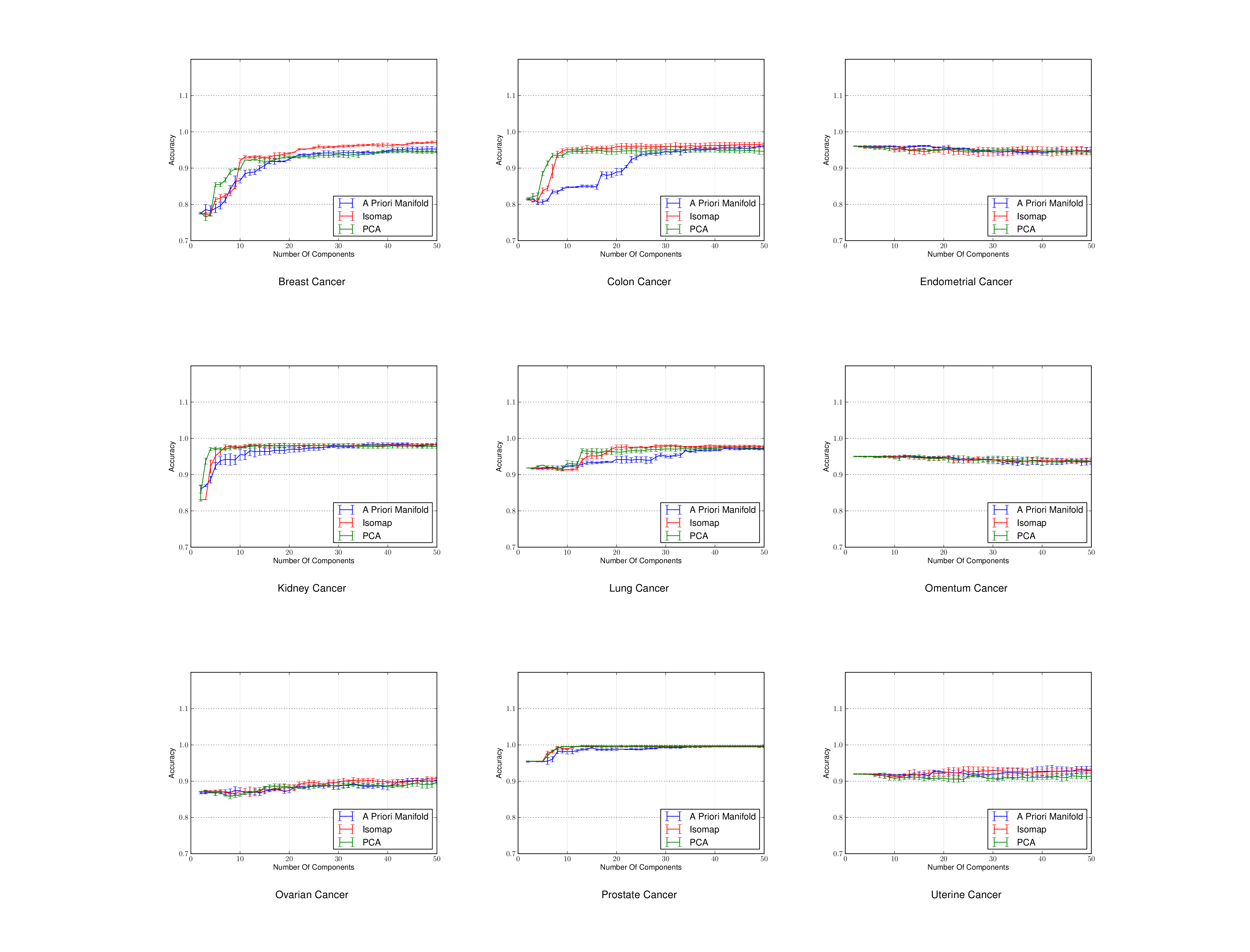

Supplement: Figure S4 — Accuracy with variance for all nine datasets for sample-by-sample affinity matrices using Linear Discriminant Analysis. Accuracy with variance calculated for a priori manifold learning (blue) compared with PCA (Green) and Isomap (Red) computed using the sample-by-sample affinity matrix and the LDA classifier. (TIFF) [file pone.0090562.s004.tiff]

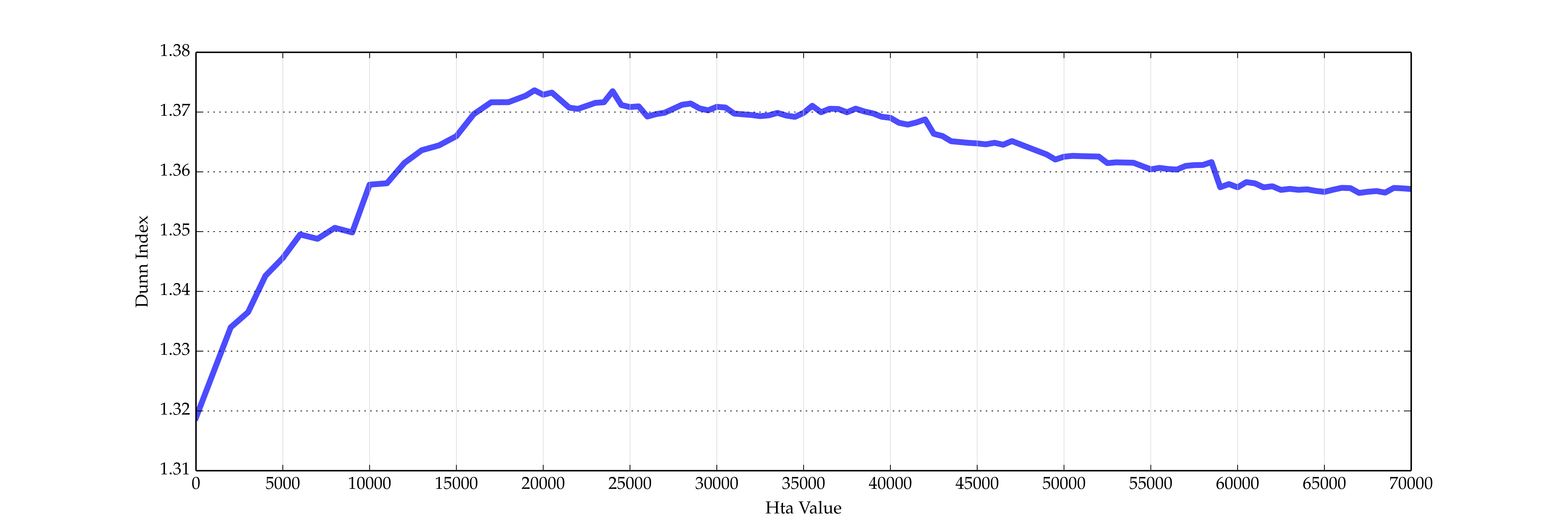

Supplement: Figure S5 — Endometrium Cancer. How the value affects the value for the Dunn Index. (TIFF) [file pone.0090562.s005.tiff]
