# Supplementary material for: An Algorithm for Finding Biologically Significant Features in Microarray Data Based on A Priori Manifold Learning
Source: PLoS One. 2014 Mar 3;9(3):e90562. doi: 10.1371/journal.pone.0090562 (PMC3940899; doi:10.1371/journal.pone.0090562)
Supplement: Material S1 — The Value. We show how the value improves the Dunn Index. The value selected for the embedding of the Endometrial cancer was 19000. It is the value with the highest Dunn Index as shown in figure S5. (PDF) [file pone.0090562.s006.pdf]

Supplementary Material 1 - The η Value

We show how the η value improves the Dunn Index. The η value selected for the embedding of the Endometrial cancer was 19000. It is the value with the highest Dunn Index as shown in figure S1

Figure S1. Endometrium Cancer: How the η value affects the value for the Dunn Index
