# Supplementary material for: An Algorithm for Finding Biologically Significant Features in Microarray Data Based on A Priori Manifold Learning
Source: PLoS One. 2014 Mar 3;9(3):e90562. doi: 10.1371/journal.pone.0090562 (PMC3940899; doi:10.1371/journal.pone.0090562)
Supplement: Material S2 — Accuracy Variance. We present the error bars with one standard deviation of uncertainty for the 10-fold cross validation with a k-NN classifier in figure S2 for the sample-by-sample affinity matrix and in figure S1 for gene-by-gene affinity matrix. For Linear Discriminant Analysis the gene-by-gene errorbars are shown in figure S3 and for the sample-by-sample experiments in figure S4. (PDF) [file pone.0090562.s007.pdf]

Supplementary Material 2 - Accuracy Variance

We present the error bars with one standard deviation of uncertainty for the 10-fold cross validation with a k-NN classifier in figure S1 for the sample-by-sample affinity matrix and in figure S2 for gene-by-gene affinity matrix. For Linear Discriminant Analysis the gene-by-gene errorbars are shown in figure S4 and for the sample-by-sample experiments in figure S3

Figure S1. Accuracy with variance for all nine datasets for sample-by-sample affinity matrices using k Nearest Neighbours:
Accuracy with variance calculated for *a priori* manifold learning (blue) compared with PCA (Green) and Isomap (Red) computed using the sample-by-sample affinity matrix and the k-NN classifier

Figure S2. Accuracy with variance for all nine datasets for gene-by-gene affinity matrices k Nearest Neighbours:
Accuracy with variance calculated for *a priori* manifold learning (blue) compared with PCA (Green) and Isomap (Red) computed using the gene-by-gene affinity matrix and the k-NN classifier

Figure S3. Accuracy with variance for all nine datasets for sample-by-sample affinity matrices using Linear Discriminant Analysis:
Accuracy with variance calculated for *a priori* manifold learning (blue) compared with PCA (Green) and Isomap (Red) computed using the sample-by-sample affinity matrix and the LDA classifier

Figure S4. Accuracy with variance for all nine datasets for gene-by-gene affinity matrices using Linear Discriminant Analysis:
Accuracy with variance calculated for *a priori* manifold learning (blue) compared with PCA (Green) and Isomap (Red) computed using the gene-by-gene affinity matrix and the LDA classifier
